# Age, Attitudes to Aging, and Identity in Older Canadian Women

**DOI:** 10.1093/geroni/igab046.213

**Published:** 2021-12-17

**Authors:** Nicky Newton

**Affiliations:** Wilfried Laurier University, Waterloo, Ontario, Canada

## Abstract

The life course perspective emphasizes social structure, personal agency, and their interdependencies (Settersten et al., 2020), serving as the theoretical framework for this study. Given stereotypical societal views of gender and aging (e.g., Sontag, 1979), physical aging is often the focus when examining women’s aging attitudes and concomitant changes in a sense of personal identity. Additionally, studies of midlife women have found relationships between age and identity (e.g., Stewart et al., 2001). Using quantitative and qualitative data, the present study examines associations between age, personal identity, and attitudes to physical, psychological and social aging in older Canadian women (N = 190, Mage = 70.38). Results show that while attitudes to physical aging contribute to identity maintenance, attitudes to social and psychological aging are also important for older women’s identity maintenance. Interactions between age and attitudes to aging associated with personal identity are discussed with reference to the life course perspective.

